# Successful in vitro fertilization following conservative surgery for synchronous endometrioid tumor of ovary and uterus

**DOI:** 10.1186/s13048-023-01137-x

**Published:** 2023-03-29

**Authors:** Vanita Suri, Ramandeep Bansal, Neelam Aggarwal, Pooja Sikka, Seema Chopra, Subhash Chandra Saha, Nalini Gupta, Bhavana Rai

**Affiliations:** 1 Department of Obstetrics and Gynecology, Post Graduate Institute of Medical Education and Research, Chandigarh, India; 2 Department of Cytology and Gynaecological Pathology, Post Graduate Institute of Medical Education and Research, Chandigarh, India; 3 Department of Radiotherapy, Post Graduate Institute of Medical Education and Research, Chandigarh, India

**Keywords:** Synchronous endometrial and ovarian cancer, Successful pregnancy outcome, in vitro fertilization, Chemotherapy

## Abstract

**Background:**

Successful pregnancy outcome in women with synchronous ovarian and endometrial cancers is very rare. We report successful pregnancy outcome in a young woman managed conservatively for synchronous endometrial and ovarian cancer.

**Case presentation:**

Thirty years old nulliparous lady presented following exploratory laparotomy, left salpingo-oophorectomy and hysteroscopic polypectomy for left adnexal mass. Histology revealed endometrioid carcinoma of left ovary and moderately differentiated adenocarcinoma in the resected polyp. She underwent staging laparotomy along with hysteroscopy which confirmed above findings without any evidence of further tumor spread. She was treated conservatively with high dose oral progestin (megestrol acetate, 160 mg) and leuprolide acetate 3.75 mg monthly injections for three months along with four cycles of carboplatin and paclitaxel based chemotherapy followed by monthly injection of leuprolide for further three months. After failure of spontaneous conception, she underwent ovulation induction for six cycles along with intrauterine insemination which failed. She underwent in vitro fertilization with donor egg followed by elective cesarean section at 37 weeks of gestation. She delivered a healthy baby of weight 2.7 kg. Intraoperatively 5 × 6 cm right ovarian cyst was found which drained chocolate coloured fluid on puncture and cystectomy was carried out. Histological examination revealed endometrioid cyst of right ovary. Uterus was spared as she wanted to preserve her fertility. She is being followed periodically and is normal nine months following delivery. She is on injection Depot medroxy progesterone acetate once every three months.

## Background

Synchronous ovarian and endometrial cancer defined as simultaneous occurrence of cancers in ovary and uterine endometrium, though not uncommon, is encountered only rarely in clinical practice [1]. We report a young woman with synchronous endometrial and ovarian cancer who was managed conservatively and had a successful pregnancy outcome.

## Case presentation

Thirty years old nulliparous lady presented three and a half years back (November 2016) to our tertiary care hospital. She had already been evaluated at another center for abnormal vaginal bleeding and found to have left adnexal mass. Subsequently she underwent exploratory laparotomy, left salpingo-oophorectomy and hysteroscopic polypectomy. Postoperative histopathological examination revealed endometrioid carcinoma of left ovary and moderately differentiated adenocarcinoma in the resected polyp. Omental and peritoneal biopsies did not reveal evidence of tumor deposits. Two weeks after surgery, she presented to our tertiary care hospital for further evaluation. Histopathological review of slides confirmed the previous findings. She was diagnosed as a case of synchronous endometrioid carcinoma of ovary and adenocarcinoma of uterus and was further evaluated. Detailed general physical, systemic and gynecological examinations were normal.

Routine hematological, biochemical, hormonal profile was normal and tumor markers were not elevated (Table 1).

**Table 1:** Hematological, biochemical and hormonal profile of index patient

Blood test	Value
Hemoglobin	10 g/dL
Total leucocyte count	7200/ µL
Differential leucocyte count	Neurtophils-76%; Lymphocytes- 24%
Platelet count	256,000/ µL
Erythrocyte sedimentation rate	22 mm/ 1^st^ hour
Random blood sugar	89 mg/ dL
Blood urea	32 mg/dL
Serum creatinine	0.9 mg/ dL
Serum bilirubin	1.1 mg/ dL
SGOT/ SGPT/ ALP	21/22/178 IU/ L
Serum albumin/ globulin	4.3/2 g/ dL
Serum calcium/ phosphorus	8.7/ 3.8 mg/ dL
Serum sodium/ potassium	133/ 3.8 mEq/ dL
Serum Luteinising hormone	11.1 IU/L
Serum follicular stimulating hormone	3.2 IU/L
Serum anti mullerian hormone	3.7 ng/ mL
Serum carcinoembryonic antigen	1.5 µg/L
Serum alpha feto protein	12 ng/mL
Serum CA 19–9	22 U/ mL
Serum CA- 125	21U/ mL
Serum inhibin	19 pg/ mL
Serum beta human chorionic gonadotropin	3Miu/ mL
Serum T3/ T4/ Thyroid stimulating hormone	122 ng/ dL; 1.4 ng/dL; 4.2 mIU/L

Abb: *SGOT* Serum glutamic oxaloacetic transaminase, *SGPT* Serum glutamic pyruvate transaminase, *ALP* Alkaline phosphatase

**Fig. 1:**
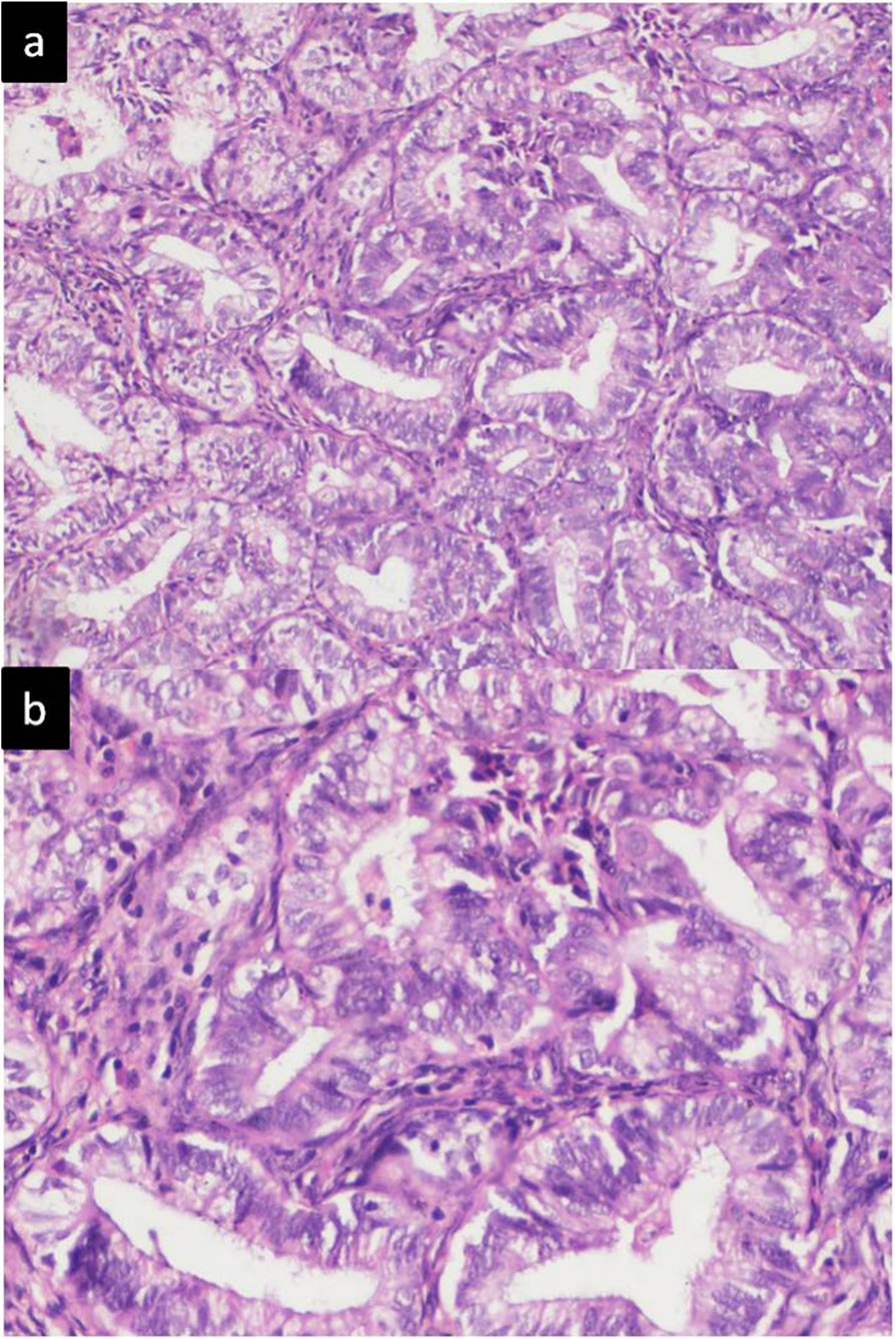
a: Endometrioid adenocarcinoma of the ovary showing complex glandular pattern and desmoplastic stroma (H&E x20X). b: The individual glands are lined by tall columnar lining epithelial cells showing moderate pleomorphism and prominent nucleoli (H&E x40X)

Gadolinium enhanced magnetic resonance imaging of pelvis revealed two tiny hemorrhagic cysts in right ovary. There was no focal lesion in the uterine cavity and endometrial thickness was 5 mm. She underwent staging laparotomy along with hysteroscopy. Intra-operatively right ovary and fallopian tube were found to be normal while left ovary and fallopian tube were absent. No metastatic deposits were found in any abdominal or pelvic organs. Biopsies were taken from right ovary, omentum and lymph nodes along with peritoneal washing. Hysteroscopy guided uterine curetting was done in the same setting. Frozen sections obtained during the procedure did not reveal any evidence of tumor. Histopathological examination of uterine curetting revealed moderately differentiated adenocarcinoma (Fig. 1). She was planned for conservative management as she desired preservation of fertility. She was given high dose oral progestin (megestrol acetate, 160 mg) and leuprolide acetate 3.75 mg monthly injections for three months along with four cycles of carboplatin and paclitaxel based chemotherapy as per our protocol (Table 2).

**Table 2:** Routine evaluation and Management protocol in our institute for synchronous endometrial and ovarian cancer for fertility preservation

Complete hemogram (Hemoglobin, total and differential leucocyte counts, platelet counts, erythrocyte sedimentation rate and C reactive protein)
Complete biochemistry (Renal, liver and thyroid function tests; serum calcium and phosphorus; serum electrolytes)
Tumour markers (alpha feto protein, carcinoembryonic antigen, CA-125, CA 19–9, Inhibin and Beta- human chorionic gonadotropin
Hormones: Luteinising hormone, follicle stimulating hormone and antimullerian hormone levels
Ultrasonography abdomen and pelvis/ contrast enhanced computed tomographic (CECT) of chest, abdomen and pelvis/ Gadolinium enhanced magnetic resonance imaging of abdomen/ pelvis
Hysteroscopic endometrial biopsy followed by histo-pathological examination Fine needle aspiration cytology of ovarian mass based on radiological findings and operability
Fertility sparing surgery (unilateral salpingo-oophorectomy ± contralateral cystectomy) with preservation of uterus
High dose oral progestin (megestrol acetate, 160 mg) twice daily with or without Intravenous leuprolide acetate 3.75 mg once a month for six months;
Hysteroscopy biopsy and imaging at three monthly intervals for six months and thereafter at 3–6 months depending upon results of biopsy; In non-responders, completion surgery is performed
Chemotherapy (Carboplatin and paclitaxel) once every 3 weeks for 3–6 cycles
Follow up for ovarian cancer- CA 125 and CECT abdomen and pelvis once every three months
Further management based on results of follow up

After three months she underwent hysteroscopy guided endometrial biopsy which was normal. She was given monthly injection of leuprolide for further three months. Repeat CA-125 levels were normal.

Six months later, she was allowed for spontaneous conception. Her cycles were monitored along with ovulation study. A repeat hysteroscopy guided endometrial biopsy was normal. However she did not conceive spontaneously and was planned for assisted reproduction one year after last cycle of chemotherapy. Routine ultrasonography revealed right ovarian endometriotic cyst. Prior to assisted reproduction she was planned for diagnostic hystero-laparoscopy, but she did not agree for the same. Hysteroscopy guided endometrial biopsy did not reveal any abnormality. As she wanted conception despite explaining the risks, she underwent ovulation induction for six cycles along with intrauterine insemination. However she still did not conceive. She was planned for right sided oophorectomy followed by in vitro fertilization (IVF) with donor egg subject to absence of any abnormality in right ovary. However she did not agree for procedure and underwent IVF with donor egg at another center. She again presented to us at 27 weeks of gestation along with cholestasis of pregnancy for which she was managed conservatively. She underwent elective cesarean section at 37 weeks of gestation and delivered a healthy baby of weight 2.7 kg. Intraoperatively, 5 × 6 cm right ovarian cyst was found which drained chocolate coloured fluid on puncture and cystectomy was carried out. Histological examination revealed endometriotic cyst of right ovary. Uterus was spared as she wanted to preserve her fertility. She is being followed periodically and is normal nine months following delivery. She is being administered injection depot medroxy progesterone acetate once every three months.

## Discussion

Simultaneous occurrence of ovarian and uterine endometrial cancer is relatively uncommon. Approximately 3–10% of women with ovarian cancer have simultaneous endometrial cancer while 3–5% of women with cancer of uterine endometrium have simultaneous cancer of ovary [1–4].

In one study [5] (*n* = 235,454 women with endometrial carcinoma), synchronous ovarian cancer was found in 1.7% of women with endometrial carcinoma, the incidence being highest (3.1–5%) in women between 29–47 years of age. The women with synchronous endometrial carcinoma and ovarian cancer were more often young black women with grade 1–2 tumors of endometrioid or serous histology type and stage I-II disease [5]. Most common histological combination was endometrioid cancer in both ovary and uterus (45.6%) followed by endometrioid cancer in uterus and non-endometrioid cancer in ovary (33%) and non-endometrioid cancer in both ovary and uterus (16.1%). Women with synchronous endometrioid cancer in both ovary and uterus were younger and had better 10 year survival rate (88.7%) compared to women with endometrioid carcinoma of uterus and non-endometrioid cancer of ovary (74.2%), non endometrioid cancer in both ovary and uterus (62.9%) and women with only endometrial carcinoma (80.7%) [5]. In another study [2], 3.3% of women with endometrial cancer and 2.7% of women with cancer of ovary had evidence of synchronous cancer of ovary or uterus respectively.

The exact mechanisms for this association remain to be determined. One possible explanation may be related to the fact that molecular receptors present in epithelial lining of ovaries and uterus are shared. Their concurrent response to a common carcinogenic insult in an index patient may lead to development of synchronous cancers in uterus and ovary [2]. For instance, Cheng et al. [6] found that epithelial ovarian cancers show aberrant expression of HOX genes that control mullerian duct differentiation and are expressed in endometrial epithelium as well. genes can result in synchronous endometrial and ovarian cancers. HOX genes are extremely sensitive to change in hormones (estrogens and progestogens) in peritoneal cavity to which ovarian surface epithelial cells are exposed. Accordingly aberrant expression of HOX.

The common clinical symptoms of synchronous ovarian and uterine cancer include abnormal vaginal bleeding (41%), pain (21%) or fullness (18%) in abdomen [2]. The symptoms which can be attributed to uterine carcinoma usually dominate the symptoms. In fact presence of abnormal vascular bleeding in setting of endometrioid ovarian cancer suggests coexistent endometrial pathology as was the case in our patient. In one study [7], 10/11 (90.9%) of women with abnormal vaginal bleeding with endometrioid ovarian tumor had coexistent pathology which is much higher than general population (1.3% of premenopausal [8] and 17.1% of menopausal women [9] with abnormal vaginal bleeding harbor endometrial cancer or atypical hyperplasia). Women with abnormal vaginal bleeding are detected and treated earlier as they seek medical help. The median survival of women with synchronous ovarian and uterine cancer was 60 months in presence of abnormal vaginal bleeding compared to 54 months in its absence in one study [2], likely related to early detection and treatment of former group.

Synchronous ovarian and uterine carcinomas are usually characterized by low histological grade and early disease stage [2]. Staging of disease in women with synchronous cancers of ovary and uterus poses difficulties especially in women with similar histology at both sites. In conveyance with cancer staging, such a situation may qualify either for stage III uterine cancer or stage IIA ovarian cancer, instead of qualifying for synchronous primarily independent cancers. However the prognosis of patients with synchronous ovarian and uterine cancers is better than either of stage III endometrial or stage IIA ovarian cancer, suggesting synchronous cancers to be an independent entity [2].

Recommended treatment for management of endometrial adenocarcinoma is total abdominal hysterectomy with bilateral salpingo-oophorectomy ± pelvic and para-aortic lymphadenectomy [10]. However, such an approach may not be feasible in women desiring future fertility. The universally accepted indications for fertility preserving treatment include strong desire for preserving fertility, nulliparous status, endometrioid carcinoma, stage I disease, reliable follow up, normal serum CA125 and grade I (grade II in some studies) histology [10]. Our patients qualified for all these criteria and were chosen for fertility preserving treatment with high dose progesterone therapy as reported in another study [11].

Similar to endometrial carcinoma, management of endometrioid ovarian tumors requires special considerations in women who desire future fertility. Though radical surgery (bilateral salpingo-oophorectomy with hysterectomy and staging surgery) remains the treatment of choice for ovarian malignancies, it may not be possible in women who desire to preserve their fertility. Accordingly in this subgroup, conservative approach has to be adopted [4]. In one review [12] including 507 women undergoing fertility sparing surgery for early-stage ovarian cancer, risk of recurrence was 10.3% and risk of death from disease was 5.5%. These values were similar to historical controls. 36.7% of these women had full term deliveries. However other series have reported recurrence rate of 4–6% in preserved ovary [13, 14]. Morice et al. [15] concluded that stage IA grade 1 and possibly grade 2 tumors (serous, mucinous or endometrioid subtypes) can be considered for fertility sparing surgery. They also suggested that even women with Grade 1 stage 1C disease can be considered for surgery.

Overall only 1/3^rd^ of women with cancers of ovary are able to achieve full term pregnancy following fertility sparing surgery [12]. The reasons for poor fertility in these women are not clear but may be related to coexistent endometrial pathology and adhesions and alteration of ovarian function after surgery. Our patient also did not conceive spontaneously and even after treatment for infertility. However she did conceive following in vitro fertilization and had successful pregnancy outcome.

While fertility sparing treatment is well documented in patients with either endometrial or ovarian carcinoma, reports of fertility sparing treatment are sparse in literature with regards to synchronous cancers of ovary and uterus [16]. Atallah et al. [16] reported a lady with synchronous ovarian and uterine cancer who conceived spontaneously and had a successful pregnancy outcome following fertility sparing treatment. However unlike their report, our patient could not conceive spontaneously and needed in vitro fertilization.

## Conclusion

Above review and our report throw further light on some of the diagnostic and therapeutic aspects related to synchronous endometrioid tumors of ovary and uterus. A rational therapeutic approach, close follow up and careful planning taking into account requirements of the patient remain keys to successful management of these rare tumors. A conservative management followed by active fertility planning may be required for women who desire fertility.

## Data Availability

All data generated or analysed during this study are included in this published article. Furthermore, the datasets during and/or analysed during the current study is also available from the corresponding author whenever requested.
